# Prevalence of fibrodysplasia ossificans progressiva (FOP) in France: an estimate based on a record linkage of two national databases

**DOI:** 10.1186/s13023-017-0674-5

**Published:** 2017-06-30

**Authors:** Geneviève Baujat, Rémy Choquet, Stéphane Bouée, Viviane Jeanbat, Laurène Courouve, Amélie Ruel, Caroline Michot, Kim-Hanh Le Quan Sang, David Lapidus, Claude Messiaen, Paul Landais, Valérie Cormier-Daire

**Affiliations:** 1 Institut Imagine, Centre de Référence Maladies Osseuses Constitutionnelles, Université Paris Descartes-Sorbonne Paris Cité, Hôpital Necker-Enfants malades, 149 rue de Sèvres, 75015 Paris, France; 2 BNDMR, Assistance Publique Hôpitaux de Paris, Hôpital Necker Enfants Malades, F-75015 Paris, France; 3 INSERM, UPMC Université Paris 06, UMR_S 1142, LIMICS, F-75006 Paris, France; 4 CEMKA, Epidémiologie, 43 boulevard du Maréchal Joffre, 92340 Bourg La Reine, France; 5 LapidusData Inc., Oklahoma City, OK USA; 6 UPRES EA2415, Clinical Research University Hospital, Montpellier University, Montpellier, France; 7 BESPIM, Nimes University Hospital, Nîmes, France

**Keywords:** Fibrodysplasia ossificans progressiva, Epidemiology, Prevalence, Data bases, Rare genetic diseases

## Abstract

**Background:**

Fibrodysplasia ossificans progressiva (FOP) is a rare, severely disabling, and life-shortening genetic disorder that causes the formation of heterotopic bone within soft connective tissue. Previous studies found that the FOP prevalence was about one in every two million lives. The aim of this study is to estimate the FOP prevalence in France by probabilistic record-linkage of 2 national databases: 1) the PMSI (*Programme de médicalisation des systèmes d’information*), an administrative database that records all hospitalization activities in France and 2) CEMARA, a registry database developed by the French Centres of Reference for Rare Diseases.

**Results:**

Using a capture-recapture methodology to adjust the crude number of patients identified in both data sources, 89 FOP patients were identified, which results in a prevalence of 1.36 per million inhabitants (CI95% = [1.10; 1.68]). FOP patients’ mean age was 25 years, only 14.9% were above 40 years, and 53% of them were males. The first symptoms – beside toe malformations- occurred after birth for 97.3% of them. Mean age at identified symptoms was 7 years and above 18 years for only 6.9% of patients. Mean age at diagnosis was 10 years, and above 18 years for 14.9% of the patients. FOP patients were distributed across France.

**Conclusions:**

Despite the challenge of ascertaining patients with rare diseases, we report a much higher prevalence of FOP in France than in previous studies elsewhere. We suggest that efforts to identify patients and confirm the diagnosis of FOP should be reinforced and extended at both national and European level.

## Background

Fibrodysplasia ossificans progressiva (FOP, OMIM #135100) is a rare, severely disabling and life- shortening genetic disorder characterized by the formation of heterotopic bone within soft tissues [1]. Bilateral congenital malformations of the great toes, typically short and bent inward, are a hallmark symptom of FOP; when coupled with postnatal progressive heterotopic endochondral ossifications, this sign provide a convincing rationale for a clinical diagnosis of FOP. The FOP gene was discovered in 2006 [2], and molecular testing is now available in many countries to confirm a clinical diagnosis of FOP. During the first decade of life, patients experience episodes of painful soft tissue swellings (called flare-ups), with most such flare-ups resulting in conversion of soft tissue to anatomically normal bone deposited in ectopic locations, a process known as heterotopic ossification (HO). The HO process is progressive and cumulative. HO typically begins in the dorsal, axial, cranial, and proximal regions of the body, and later involves in the ventral, appendicular, caudal and distal regions [3]. HO develops into segments, sheets, and ribbons of extra bone throughout the body and across joints, thereby progressively restricting movement. Flare-ups may occur spontaneously or be precipitated by injury, intramuscular injections, immunizations, biopsies or surgeries, viral infection, muscular stretching, falls, or fatigue. By the second decade, ankylosed joints cause many affected individuals to lose mobility. By third decade, many are wheelchair-bound and require caregiver assistance to perform daily living activities. By the fourth decade, many patients are at risk of early death due to thoracic insufficiency syndrome [4, 5] or thrombosis.

FOP is caused by a mono-allelic gain of function mutation in the activin receptor type IA (*ACVR1*), also known as activin like kinase 2 (ALK2) type I receptor [6]. The vast majority of cases are due to the same alteration, R206H, and results from a spontaneous de novo mutation [7]. A paternal age effect has been reported [8]. Previous studies in Spain and the United Kingdom estimated FOP prevalence at about one case per two million population [9, 10]. FOP has no ethnic, gender or geographic predisposition.

The aim of this study was to estimate the FOP prevalence in France, by cross-linking 2 national databases: First, the PMSI (*Programme de médicalisation des systèmes d’information*), which began operation in France in 1986. Its use became mandatory in all public and private health facilities in 2005 to receive reimbursement for medical services [11]. Second, the CEMARA database (“*CEntres de référence MAladies RAres*”, Rare Diseases (RD) Centres of Reference for rare diseases) which was initiated as part of the first National Plan for Rare Diseases launched in 2004 [12]. The CEMARA system records epidemiological and diagnostic data of RD patients refered to the Centres of Reference and related networks, either at out-patient clinics or during hospitalizations.

## Methods

### Material

Our study extracted and matched data from the two above mentioned health datasets, CEMARA and PMSI. CEMARA is a non - population - based registry that was launched in 2007. It collects information on rare diseases’ epidemiology and related medical activities [12] from rare diseases Centers of Reference (CR) in France. Its goal is to improve the understanding of the burden of disease for rare conditions as well as the resources needed to treat those conditions. CEMARA also helps identify patients who might be eligible for natural history studies and clinical trials. Since 2007, a minimum data set has been collected from all RD patients who are diagnosed, followed, and treated at designated national CRs. The minimum dataset includes administrative and medical data such as: date of birth, place of birth, place of residency, sex, diagnosis, familial or sporadic occurrence, diagnostic confirmation method (clinical, molecular or other), and date of death. FOP-related information captured by this database has been validated by the CR-MOC (Centre de Référence des Maladies Osseuses Constitutionnelles; reference center for constitutional rare bone diseases). Participants are physicians involved in diagnostic procedures (including medical geneticists, paediatricians, rheumatologists) or management (including orthopaedists, endocrinologists, psychologists, genetic counsellors, social workers). The system allows longitudinal tracking of individual patients.

The Programme de Médicalisation des Systèmes d’Information (PMSI) [medical information system program] is an administrative database that captures two main types of healthcare events [13]. First, it includes full coverage of all hospital admissions in Medicine, Surgery, and Obstetrics (Médecine Chirurgie et Obstétrique, MCO). The MCO also includes day hospitalizations which are less than 24 h’ duration (no overnight stay). Outpatients who receive only consultations, or radiological or other examinations are not recorded in the PMSI. The second type of events captured in PMSI – via the SSR (*Soins de Suite et de Réadaptation;* readaptation and rehabilitation centers) database- is admissions to intermediate care homes/facilities, as well as readaptation and rehabilitation centers. PMSI has used longitudinal patient identifiers since 2006. The most recent data set provides coverage through 2014. The main variables in PMSI are: hospital identification number, patient identification number, patients‘ sex and age, whether the patient died after the admission, diagnoses (ICD 10–1 primary diagnosis ±1 related diagnosis and up to 20 significant associated diagnoses) [14], total duration of the patient’s stay, procedures performed during the stay, origin of the patient (home, transfer from other institution, etc.), discharge location (home, another medical unit, death). FOP patients were identified in PMSI by the presence of ICD-10 code M61.1 (Myositis ossificans progressiva or Fibrodysplasia ossificans progressiva) (http://apps.who.int/classifications/icd10/browse/2010/en#/M60-M63). However, it is essential to note that the PMSI data set has limitations: i) diagnoses are not confirmed or validated; ii) there is no narrative description of the patient’s condition or history; and iii) patients are not captured in the data set unless they are hospitalized.

### Regulatory authorizations

The study was approved by the CCTIRS (*Comité consultatif sur le traitement de l’information en matière de recherche*) [Advisory Committee on Information Processing in Material Research in the Field of Health] and by the French National Commission on Information Technology and Liberties (CNIL) (authorization number: DR-2016-048). Patients identified in the CEMARA database were individually informed of the study, including the linkage to their PMSI records, and their rights relating to the confidentiality of their data. However, since PMSI data are de-identified, patients suspected of FOP in this resource could not be individually informed of the study. For both datasets, only persons residing in France, including Metropolitan (mainland) France and overseas Departments which were present in PMSI and/or CEMARA database, were included.

### Methods

**Fig. 1:**
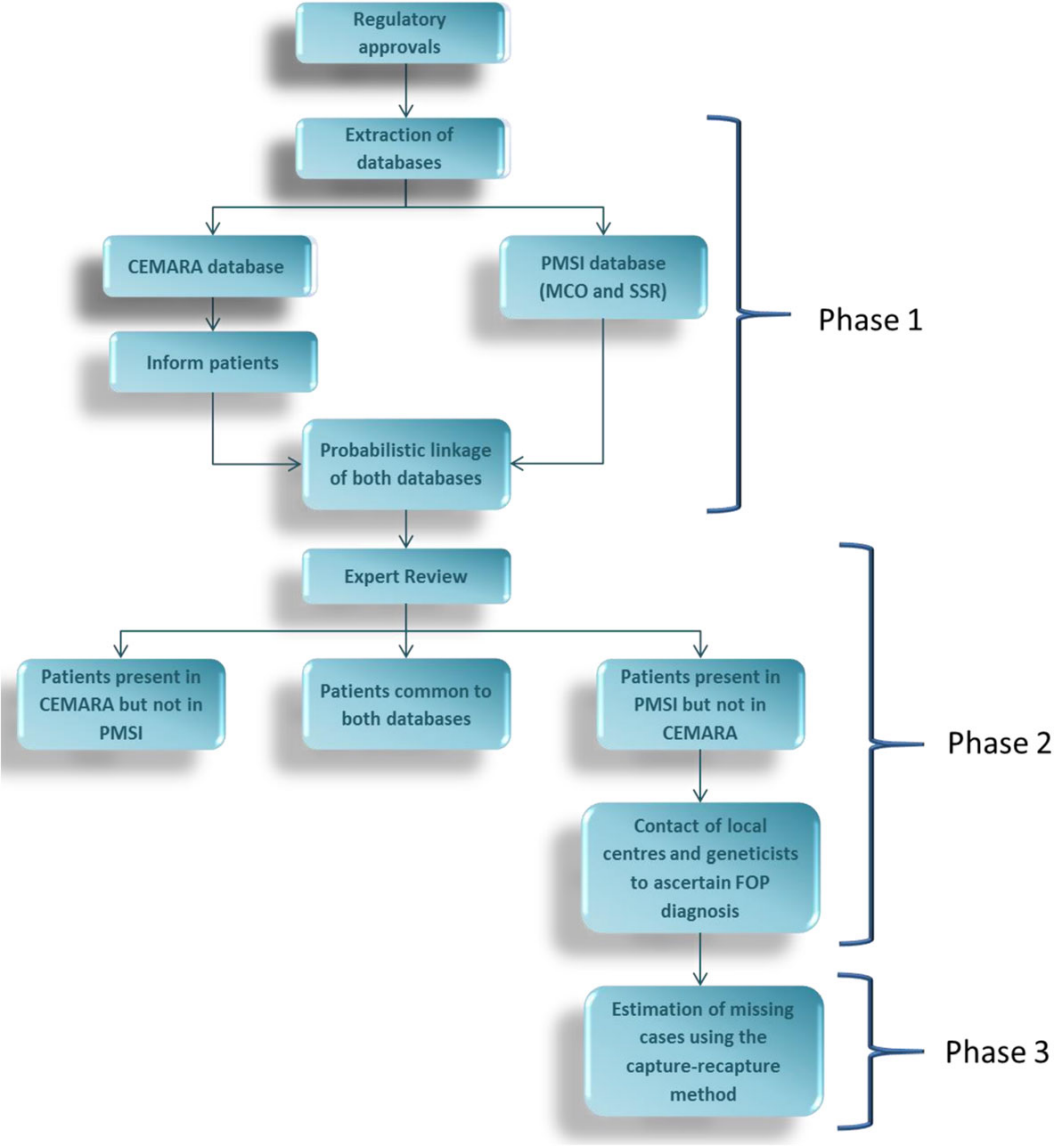
Work flow of study process

The workflow of the study is described in Fig. 1. It is composed of the following phases: 1) identification of FOP cases in PMSI and CEMARA, 2) validation of positive cases in both databases by a FOP expert from the CR-MOC and/ or its regional network and 3) estimation of missing cases using capture-recapture method.

All patients recorded in the CEMARA database were diagnosed as FOP patients by expert physicians, meaning that they were seen at least once in CR-MOC clinics, and they were diagnosed either by clinico-radiological hallmarks and/or by molecular testing, available since 2006. Patients included in both CEMARA and PMSI were considered to be true FOP patients because of their presence in the CEMARA database. PMSI records for the remaining patients (coded as FOP in PMSI but not present in the CEMARA database) were reviewed by a physician member of the CR-MOC who is an expert in FOP. After the FOP expert reviewed the patients’ PMSI records, these patients were classified as either: 1) confirmed cases of FOP; 2) false positive (claim history is incompatible with FOP); or 3) possible FOP. This review was performed by examining all of the patients’ PMSI records from the period 2006–2014. These records contain information from hospital stays, including diagnostic codes, diagnosis related groups (DRG) codes and type of care. Criteria for classifiying a patient as false positive (non-FOP) were: an older age at first hospitalization (> 60 years), and/ or the presence of another disease causing heterotopic ossifications or calcifications (for instance, peripheral arterial disease, renal failure, myositis post trauma or post hematoma, fibrous dysplasia, progressive osseous heteroplasia, dermatomyositis with calcinosis universalis, leukemia, tuberculosis). The expert then investigated each case of possible FOP by contacting the genetic team of the nearest Centre to determine whether the patient, who was present in PMSI but not CEMARA, had been evaluated in these centres/hospitals.

### Statistical analyses

Our analysis includes a capture-recapture method to estimate the prevalence of FOP cases in France as of January 1st 2012. The prevalence was calculated for this date by dividing the number of living FOP patients by the resident population. The confidence interval was calculated using Wilson’s method with a continuity correction of a Poisson law [15]. On the prevalence day, the population was 65,400,000 in Metropolitan (mainland) France and overseas Departments (https://www.insee.fr/fr/statistiques/1372599?sommaire=1372680).

The prevalence value was adjusted by estimating the true positive rate among the PMSI patients whose FOP status could not be confirmed. The adjustment was performed by applying Chao estimate [16], which is less biased if sources are independent or if cases have different probabilities of being included in both sources [17]. Our capture-recapture methodology uses two sources, so the unbiaised estimator of cases can be calculated as:

$$ \widehat{N}=\frac{\left(\mathrm{n}1+1\right)\left(\mathrm{n}2+1\right)}{\left(\mathrm{f}2+1\right)}-1 $$

where n1 is the number of confirmed cases in the *first* source, n2 the number of confirmed cases in the *second* source and f2 is the number of common cases.

Statistical analysis was performed with SAS software, version 9.3 (North Carolina, USA).

## Results

**Fig. 2:**
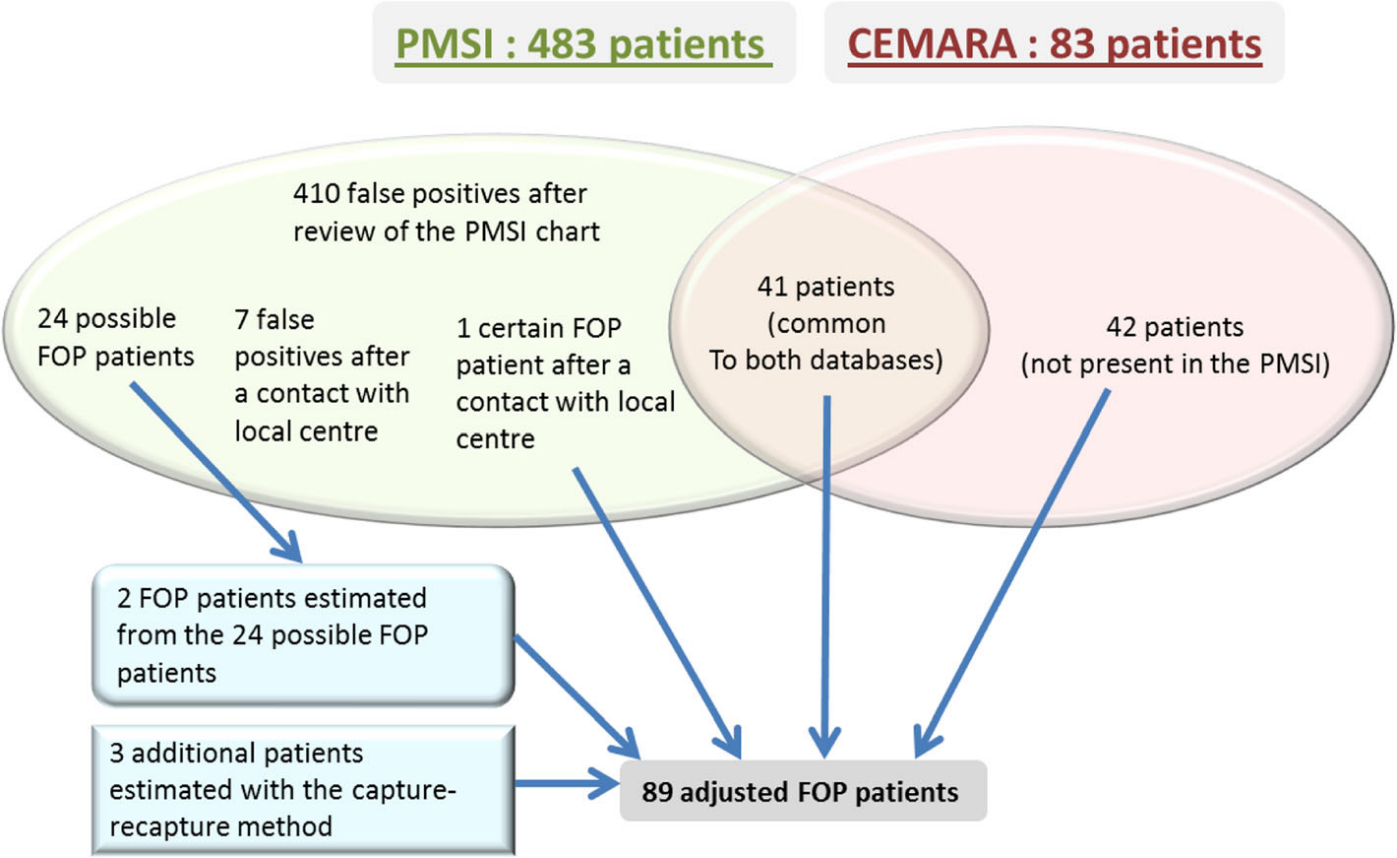
Result of the linkage of the 2 databases

The number of FOP patients in both databases are reported in Fig. 2 and Table 1. In the PMSI and CEMARA databases, respectively 483 and 83 patients have been identified. Among these patients: 1) 41 patients were present in both databases and are all true positive FOP patients; 2) 42 patients were present in the CEMARA database but not in the PMSI and are all true positive FOP patients; and 3) 442 patients were in the PMSI database but not in the CEMARA database. Among the 442 PMSI patients who were not present in CEMARA, 410 were determined to be false positives after the review of their PMSI records, while 32 were still suspected cases, whose history was compatible with FOP but not confirmatory. For these 32 cases, our FOP expert contacted local genetic teams and confirmed 1 patient as a true positive case of FOP and eliminated 7 cases as false positives (not FOP). However, the remaining 24 cases could not be confirmed or eliminated. Thus, among the 483 patients in PMSI with FOP diagnosis codes, 417 were false positives and 42 were true positives.

**Table 1:** Result of the linkage of the 2 databases

	PMSI			Common patients	CEMARA	
	483 ICD-10 coded patients				83 Orphacoded patients	
Validation/correction method	False positives	Possible FOP	True positives		True positives	False positives
Medical expert	410	32	41	41	83	0
Healthcare local center review of possible FOP cases	7	24	1			
Classification of 24 possible FOP cases	22	0	2			
TOTAL before capture-recapture	439	0	44	41	83	0
Estimation of missing cases outside the two datasets (Chao)	3					
TOTAL adjusted FOP cases	89					

The proportion of the 24 remaining patients who are true positive cases was estimated to be 9.2% (42 positive FOP cases in the PMSI divided by the 459 patients for whom the FOP diagnosis could be stated). Therefore, 2 patients (24*9.2%) were added to the estimate of true positive FOP cases in PMSI. Finally, using the capture-recapture Chao estimate, the population of FOP patients is 89. The estimated prevalance in France is 1.36 per million inhabitants (CI95% = [1.10; 1.68]).

Table 2 describes the subjects found in CEMARA and/or PMSI. The mean age of FOP patients was 25 years, only 14.9% were above 40 years, and 53% of them were males. The first symptoms – beside the toe malformations- occurred after birth for 97.3% of the patients. Mean age at first symptoms, besides the hallux valgus, was 7 years. It occurred above 18 years in only 6.9% of cases. Mean age at diagnosis was 10 years and above 18 years in 14.9% of cases. The geographical distribution of FOP patients did not show a specific pattern and seemed to be randomly distributed in whole France.

**Table 2:** Patients characteristics

	Total
Number of FOP patients in CEMARA and/or PMSI	84
Gender	
MD*	1
Male	44 (53.0%)
Female	39 (47.0%)
Age on January 1, 2012 (years)	
MD	3
10 years and less	18 (22.2%)
11 to 20	20 (24.7%)
21 to 30	13 (16.0%)
31 to 40	17 (21.0%)
41 to 50	5 (6.2%)
51 to 60	5 (6.2%)
60 and older	3 (3.7%)
Age on January 1, 2012 (years)	MD = 3
Mean (standard deviation)	25.5 (16.2)
Median / Min / Max	23.0 / 2.0 / 71.0
First symptoms	
MD	10
At birth	2 (2.7%)
After the birth	72 (97.3%)
Age at onset of symptoms (years)	MD = 12
Mean (standard deviation)	7.1 (9.7)
Median / Min / Max	5.0 / 0.1 / 56.0
Age at onset of symptoms (years)	
< =1 year old	16 (22.2%)
1–2 years old	12 (16.7%)
2–4 years old	7 (9.7%)
4–6 years old	8 (11.1%)
6–8 years old	8 (11.1%)
8–17 years old	16 (22.2%)
> =18 years old	5 (6.9%)
Age at diagnosis (years)	MD = 17
Mean (standard deviation)	10.2 (12.9)
Median / Min / Max	6.4 / 0.5 / 58.0
Age at diagnosis (years)	MD = 17
< =1 year old	9 (13.4%)
1–2 years old	9 (13.4%)
2–4 years old	10 (14.9%)
4–6 years old	5 (7.5%)
6–8 years old	9 (13.4%)
8–17 years old	15 (22.4%)
> =18 years old	10 (14.9%)

*MD* missing data

## Discussion

### Accuracy of the method used for approaching the prevalence

Our metholodology for estimating the prevalence of FOP in France was based on a capture-recapture method using 2 complementary national databases. Records were linked probabilistically between PMSI and CEMARA. PMSI is the main information system for recording the medical activity of patients in France; potential FOP patients can be identified within it by the ICD-10 diagnostic code M61.1. CEMARA is a population registry focused on rare diseases which includes FOP. Our study’s aim was to identify patients on their main care pathways, from birth to death. Through this method, the prevalence of FOP is estimated to be 1.36 per million inhabitants (or 1/735,000 births). The independence of the sources of informations could not be estimated, so we applied the Chao estimator which is more appropriate for data sources where probability of cases’ inclusion differs for each source.

### Datasources assessement

The 2 databases used in this study, CEMARA and PMSI, capture patient data in different ways that result in different coverage and quality. CEMARA captures diagnostic data from expert national centres (Centre of Reference “Maladies Osseuses Constitutionnelles”- CR-MOC) where FOP patients’ diagnosis is validated by physicians with expertise in the disease, through their out-patient or in-patient clinics. The PMSI system captures ICD diagnostic codes for in-patient clinics only.

Although the capture-recapture analysis predicts a very small number of missed cases, it is difficult to evaluate whether cases are missed from CEMARA because some patients may not reach a center of reference. Non - population - based registries, such as CEMARA, recruit from selected bodies, clinical centers or other types of structures (members of a patient organization, participants registered via an ERN – European Reference Network- or other disease-specific registry, ..) so the population coverage may not be comprehensive. Still, our study indicates the sensivity of CEMARA because only one PMSI patient was absent from CEMARA. (If the adjusted cases are included, then six PMSI patients were absent from CEMARA).

By contrast, half of the patients in CEMARA were not identified as FOP patients in the PMSI, most likely due to the fact PMSI does not capture out-patient clinic follow-up. Indeed, in the authors’experience, FOP patients are rarely hospitalized; this is typically related to patients’ impaired mobility, contraindication for surgery, and the widespread need to provide home-based care due to the severity of the disease. Our study also demonstrates the difficulty of using PMSI to identify FOP patients because of the high false-positive rate: 90% of patients with FOP codes in PMSI were either miscoded or the codes were not intended to represent the patient’s final diagnosis. Our study’s systematic review of FOP-coded patients in PMSI was based on several factors: age, main diagnosis, associated diagnosis, surgical procedures, and co-morbidity. High false-positive rates have previously been reported for conditions with heterotopic ossifications or calcifications [18]. These studies have examined the cause of these high rates (xvii): incorrect codes may be used if the patient’s disease lacks a specific ICD-10 code, the provider lacks time to identify the correct code, the provider is performing a differential diagnosis to rule out FOP, or the nominal patient is simply related to an affected FOP patient and is being seen for a family history. As an example, some bone diseases with ectopic ossifications, such as progressive osseous heteroplasia, do not have a specific ICD-10 code. Healthcare providers may use the FOP code (M61.1) in spite of its inaccuracy. Diseases with FOP as differential diagnosis include juvenile fibromatosis, desmoid tumor, chondrocalcinosis, calcinosis universalis in systemic diseases, ectopic calcifications in chronic renal insufficiency, vascular calcifications in peripheral arterial disease, ectopic ossifications after trauma hematoma or in cancer, or multiple exostosis (which may be mistaken with mild forms of FOP, particularly at early stages). Likewise, a physician may need to order diagnostic tests to rule out FOP in a patient with a tumor-like swelling; the final diagnosis could be a visually-similar condition like those cited above.

### Comparison with previous studies

Two earlier studies have estimated the prevalence of FOP. The first study was conducted in England and Wales and published in 1982 [9]. The authors surveyed several sources to estimate the total number of patients with FOP in these countries. An initial survey identified 133 potential patients with FOP, and the 3 other searches identified 3 additional patients. These 136 patients were then contacted to confirm or refute the diagnosis of FOP. Among them, 44 had a confirmed diagnosis of FOP. The other cases either had a disease other than FOP, or were not of British nationality. Of the 44 British patients with FOP, 7 medical records could not be recovered and 7 others had died. Only 30 patients could be examined, and the diagnosis of FOP was confirmed for those 30 patients. Therefore, among the 49,117,300 residents of England and Wales at the time, the 30 confirmed FOP cases yielded a prevalence of 0.61 per million.

More recently, a study in Spain examined FOP’s epidemiology [10]. The authors included a prevalence estimate of 0.36 per million in this study based on their identification of 17 patients. However, this prevalence is a minimum value because ascertainment was limited to the authors’ clinic and cases reported via passively recruited surveys.

Both of these earlier studies likely underestimate prevalence because cases were ascertained via study-specific surveys rather than established national reporting systems. These surveys are unlikely to achieve the same level of ascertainment as the present-day CEMARA and PMSI systems in France. In addition, some patients were lost to follow-up in both prior studies, which could also lower the prevalence value. These differences may explain why our estimate of FOP prevalence is higher than those in 1982 and 2012 [9, 10].

In addition to these methodological differences, the environment has changed in recent years to favor increased identification of FOP affected individuals. The internet provides access to information about FOP, and search engines allow individuals with questions about signs and symptoms to identify specific diseases that match their condition, including FOP. Also, advances in research have led to increased physician awareness of FOP and more efficient diagnostic capabilities (molecular testing), both of which assist in the identification of FOP-affected individuals, particularly in atypical forms. Finally, informatics systems such as CEMARA make it easier to conduct population studies with specific disease groups such as FOP.

### Relevance and extrapolation to other countries

Most FOP cases arise as a result of a spontaneous (de novo) mutation. Fewer than ten multigenerational FOP families are known worldwide [7]. Moreover, there are no ethnic, racial, gender or geographic predispositions ([7, 19] to FOP. Therefore, the prevalence observed in France can serve as a benchmark for extrapolation to other countries. Variation would still be expected due to differences in access to healthcare services (including in diagnostic capabilities) or differences in the efficiency of health informatics systems.

### Why was this study conducted in France?

France has three important characteristics that make it a good environment for population studies: 1) The healthcare system permits a thorough ascertainment of rare diseases like FOP through a centralized pathway of care for complex and chronic rare diseases (specific Centres of Reference organization); 2) the Centre of Reference network systematically records patient data, including diagnosis and place of residence, for patients with rare diseases in the CEMARA database; and 3) the national healthcare system covers a population large enough to yield a narrow confidence interval for the prevalence estimate. France’s healthcare system uses a referral network with one national Centre of Reference and 14 regional Centres of Competence to diagnose and treat patients with rare skeletal dysplasias. The role of the local and community physicians is to refer suspected patients to these Centres at least once to ensure appropriate diagnosis and management. Because diagnosis and treatment are centralized in a relatively small number of facilities, ascertainment of FOP cases in France is likely to be more thorough compared to a country with a less-structured referral system. Nearly all PMSI patients are also found in CEMARA, which is an independent indicator of the system’s success in referring FOP patients to the expert network. Other countries, such as the Netherlands and Scandinavian countries (among others), have also produced reliable assessments of rare disease prevalence. These countries’ relatively small populations and highly structured referral networks support the ascertainment of patients for epidemiology studies [20–23]. Our study demonstrates that similar outcomes can be achieved in a larger country where initiatives such as CEMARA and CR-MOC have been implemented. Finally, our study demonstrates the utility of national daata-capture mechanisms in dealing with the challenges of rare disease epidemiology, including the small number of cases and their heterogeneity. As with CEMARA, a data capture system is more valuable for epidemiological analysis and case ascertainment if it is linked to other national initiatives (such as PMSI).

### Future research

Several sources can be used to ascertain phenotypic patients: patient groups, healthcare providers, registries, administrative databases, health records (digitally or manually), and others. Each source has limits: many can only identify patients who have already been diagnosed (which represents a selection bias), and administrative databases can yield false positives because diagnosis codes are not validated. Our study combines several sources to mitigate these disadvantages using robust capture-recapture estimators.

Another question is whether FOP prevalence may rise due to the trend toward an increased paternal age, a known risk factor for FOP as for other conditions due to single-mutation variation [8]. FOP is rarely detectable in utero. The average paternal age at conception varies considerably over time and across countries. It will be relevant to evaluate the impact that paternal age may have on genetic risks and on the prevalence of single-mutation diseases such as FOP.

This study provides basic characterization of the FOP prevalent population: age distribution, age at onset of symptoms, age at diagnosis, gender, and location of symptoms at onset of disease. Numerous other questions remain to be studied, including a detailed description of the natural history and patient-reported outcomes like quality of life and familial burden.

To better assess the epidemiology and natural history of FOP, other systems will be established in the coming years to aggregate various FOP datasets at a national and/or international levels, including the international registry project supported by IFOPA [24]. These registries could yield insights about this severe disease, including the identification of endpoints for interventional trials, development of multicentric translational research projects, and biobanking. Registries facilitate communications between health providers, research teams, biopharmaceutical companies, patient community and policy-makers. In addition, these systems could improve the understanding of FOP prevalence and its burden on patients. Finally, these registries’ goals include also the provision of better and more appropriate medical care to patients throughout France and elsewhere.

## Conclusions

This study reports a prevalence of FOP in France of 1.36 per million (or 1/ 735,000 births), which is higher than the findings of previous studies. It also describes certain elements of natural history and diagnosis. The study shows that efforts to identify diagnose and identify FOP patients should be strengthened and continued at a national and international level. Early diagnosis can prevent or delay the natural history course and avert iatrogenic complications that result from inappropriate diagnostic or treatment procedures, such as biopsy or surgery. Moreover, an improved understanding of FOP will help public health decision makers to allocate resources, educate healthcare providers, and develop treatments for this disabling disease.

## Abbreviations

CCTIRS: Comité Consultatif sur le Traitement de l’Information en matière de Recherche dans le domaine de la Santé [Consultative Committee on Data Processing in the Healthcare Research Field]
CEMARA: Centre des Maladies Rares [Centre for Rare Diseases]
CNIL: Commission Nationale de l’Informatique et des Libertés [French Data Protection Authority]
CNOM: Conseil National de l’Ordre des Médecins [National Council of the Order of Physicians]
CR-MOC: Centre de Référence – Malladies Osseuses Constitutionnelles
DRG: Diagnosis-related group
FOP: Fibrodysplasia ossificans progressiva
ICD: International classification of diseases
MCO: Médecine Chirurgie et Obstétrique [Medicine, surgery and obstetrics]
PMSI: Programme de Médicalisation des Systèmes d’Information [medical information system programme]
SSR: Soins de Suite et de Réadaptation [readaptation and rehabilitation centers]
